# A Workplace Mindfulness Intervention May Be Associated With Improved Psychological Well-Being and Productivity. A Preliminary Field Study in a Company Setting

**DOI:** 10.3389/fpsyg.2018.00195

**Published:** 2018-02-28

**Authors:** Wendy Kersemaekers, Silke Rupprecht, Marc Wittmann, Chris Tamdjidi, Pia Falke, Rogier Donders, Anne Speckens, Niko Kohls

**Affiliations:** ^1^Radboudumc Center for Mindfulness, Department of Psychiatry, Radboud University Medical Center, Nijmegen, Netherlands; ^2^Institute for Frontier Areas of Psychology and Mental Health, Freiburg, Germany; ^3^Institute of Medical Psychology, Ludwig-Maximilian University of Munich, Munich, Germany; ^4^Kalapa Leadership Academy, Cologne, Germany; ^5^Department for Health Evidence, Radboud University Medical Center, Nijmegen, Netherlands; ^6^Division of Integrative Health Promotion, University of Applied Sciences and Arts, Coburg, Germany

**Keywords:** mindfulness, workplace, intervention, meditation, stress reduction, burnout, productivity, collaboration

## Abstract

**Background:** Mindfulness trainings are increasingly offered in workplace environments in order to improve health and productivity. Whilst promising, there is limited research on the effectiveness of mindfulness interventions in workplace settings.

**Objective:** To examine the feasibility and effectiveness of a Workplace Mindfulness Training (WMT) in terms of burnout, psychological well-being, organizational and team climate, and performance.

**Methods:** This is a preliminary field study in four companies. Self-report questionnaires were administered up to a month before, at start of, and right at the end of the WMT, resulting in a pre-intervention and an intervention period. There was no separate control group. A total of 425 participants completed the surveys on the different time points. Linear mixed model analyses were used to analyze the data.

**Results:** When comparing the intervention period with the pre-intervention period, significantly greater improvements were found in measures of burnout (mean difference = 0.3, *p* < 0.001), perceived stress (mean difference = -0.2, *p* < 0.001), mindfulness [mean difference = 1.0 for the Freiburg Mindfulness Inventory (FMI) and 0.8 for the Mindfulness Attention Awareness Scale (MAAS), both *p* < 0.001], and well-being (mean difference = 0.4, *p* < 0.001). Additionally, greater increases in team climate, organizational climate and personal performance were reported during the intervention compared to the pre-intervention period with largest improvements in team cooperation (mean difference = 0.3, *p* < 0.001), productivity (mean difference = 0.5, *p* < 0.001), and stress (mean difference = -0.4, *p* < 0.001). Effect sizes were large for mindfulness (*d* > 0.8), moderate for well-being, burnout and perceived stress (*d* = 0.5–0.8), and ranged from low to moderate for organizational and team climate and personal performance (*d* = 0.2–0.8).

**Conclusion:** These preliminary data suggest that compared to the pre-intervention period, the intervention period was associated with greater reductions in burnout and perceived stress, improvements in mindfulness, well-being, and increases in team and organizational climate and personal performance. Due to design limitations, no conclusions can be drawn on the extent to which the WMT or non-specific factors such as time have contributed to the findings. Further studies, preferably using randomized controlled designs with longer follow up periods are needed to evaluate whether the associations found can be attributed to the WMT and whether these sustain after the training.

## Introduction

In an increasingly knowledge-based economy, economic value relies on skills and motivation of individuals – “human capital.” A considerable part of the workforce, however, seems to be suffering from distress and mental health issues. The European Commission (2011) reported increasing levels of work-related stress over a 10-year period. Long-term chronic stress augments the risk of suffering from mental and physical ill health. Kivimäki et al. (2015) concluded, for example, in a systematic review that working long hours enhances the risk for stroke. Moreover, an increasing number of employees experience mental health issues which are now the leading cause of sickness absence in the UK (Davies, 2014). Overall, the UK based Health and Safety Executive [HSE] (2016) estimated that 10 million working days are lost as a result of anxiety, depression and stress, which employees linked directly to work and working conditions.

There are great personal and economic costs associated with stress and resulting mental ill-health. In terms of personal costs, Wittchen and Jacobi (2005) found that the quality of life is about one standard deviation unit lower when experiencing a mental health disorder. In terms of economic costs, Andlin-Sobocki et al. (2005) concluded that the total indirect costs due to lost work days and productivity amount to 179 billion euro. Moreover, the European commission conservatively estimated that work-related stress as a sole factor accounts for annual social costs of 20 billion Euro (Levi and European Commission, 2000).

Companies are looking for ways to reduce stress and improve well-being. An intervention that would tackle the prevalence and costs associated with this problem, while improving well-being and performance would be beneficial for both employers and employees.

Chronic stress and burnout are often the result of an imbalance between job demands and an employee’s mental and physical resources (Bakker and Demerouti, 2007). For example, workload pressures and a lack of managerial support increase the risk of suffering from stress, depression and anxiety (Health and Safety Executive [HSE], 2016). However, it is important to note that there is considerable individual variability in response to work demands and acute stress which seem to be determined by a person’s coping resources.

One of the personal resources that is linked to greater coping ability is mindfulness. For example, trait mindfulness was predictive of lower psychological distress (Walach et al., 2006) and associated with lower levels of anxiety and depression and higher levels of positive affect and life satisfaction (Brown and Ryan, 2003). In a workplace setting, greater mindfulness was also linked to lower emotional exhaustion which is indicative of burnout (Hülsheger et al., 2013).

Mindfulness is defined as a state of paying attention in the present moment, on purpose and in an accepting and kind way (Kabat-Zinn, 2003). It is essentially a cognitive skill and it has been found to be improved through training. Mindfulness trainings (MTs) are typically comprised of a mixture of mindfulness practices, such as secular meditation, psycho-education and group interaction.

Over the last 20 years, MTs have often been employed to reduce stress and improve mental health in both clinical and non-clinical populations. There is now substantial evidence that MT effectively improves trait mindfulness, coping ability and psychological well-being (Chiesa and Serretti, 2009; Khoury et al., 2013; Visted et al., 2015). A comprehensive meta-analysis of 209 studies concluded that MTs compared to waitlist and active controls robustly lead to reductions in stress, anxiety and depression with moderate effect sizes (Khoury et al., 2013). However, effect sizes were not higher compared to traditional cognitive behavioral therapies (CBT) or behavioral therapies (BT).

There is also substantial evidence that changes in mindfulness mediate improvements in stress and psychological well-being (Gu et al., 2015). To illustrate, Baer et al. (2012) measured weekly changes in mindfulness and perceived stress during an 8-week mindfulness intervention. They found that mindfulness increased significantly after the 2nd week of the program, while perceived stress did not significantly change until the 4th week of the program.

However, mindfulness is more than just a way to relieve symptoms. It can be seen as part of a positive psychology movement in its capacity to improve well-being and enhance (cognitive) functioning (Fredrickson et al., 2008). To illustrate, Garland et al. (2011) showed that MT was associated with increases in positive reappraisal coping and trait mindfulness. Mindfulness and positive reappraisal seemed to mutually enhance one another leading to a positive upward spiral. In this respect, mindfulness may also be linked to improvements in work performance. Glomb et al. (2011) argued that MT in the workplace should lead to enhancements in task performance and social relationships because mindfulness improves the ability of a person to self-regulate their attention.

Good et al. (2016) developed an integrative framework relating mindfulness to workplace outcomes, identifying how mindfulness influences attention with downstream effects on cognition, emotion, behavior and physiology, ultimately impacting workplace outcomes including performance, relationships and well-being.

However, the application of mindfulness in workplace settings has just begun and there is a need for more systematic empirical research in this area. A recent systematic review identified a total of 112 studies investigating the effect of MT in the workplace (Lomas et al., 2017). It is interesting to note that the large majority of the studies included in the review were conducted in the public sector including health services and schools. However, corporate employees may have to cope with specific workplace challenges. To illustrate, presenteeism – people coming to work when being unwell – is more prevalent in the private sector (Office for National Statistics, 2014).

Potentially one of the first workplace studies in a high-stress private sector setting – a call center – was conducted by Walach et al. (2007). Twenty-three employees participated either in a waitlist control group or intervention group. They found that compared to a waitlist control group, coping strategies improved significantly in the intervention group at post-test and improvements were sustained at 3-months follow up.

Another trial in a corporate setting randomly assigned 152 middle-level managers (57% women) to an 8-week mindfulness intervention or an active control condition involving cognitive-behavioral theory and principles (Shonin et al., 2014). Strong and sustainable intervention effects were demonstrated on work-related stress, job satisfaction, psychological distress and employer-rated job performance. It was also concluded that mindfulness may be linked to more effective work styles.

Aikens et al. (2014) conducted a high-powered study in a Dow chemical company. Ninety interested employees were randomly assigned to either a control group or an intervention group. The intervention was a 7-week program combined of a first in-person class meeting, subsequent group webinar meetings of 1 h and accompanying on-line training material such as audio mindfulness exercises. Compared to the control group, the MT led to improvements in mindfulness, perceived stress and burnout. The authors additionally calculated potential company savings of up to $ 22,000 per employee, based on average wages, reductions in burnout and subsequent potential increase in workforce productivity.

To summarize, while findings are still preliminary, MTs in workplace settings show promise in improving psychological well-being and reducing stress. Studies placed in a corporate setting are largely missing. Furthermore, there is a lack of studies investigating the impact of MTs on workplace specific outcomes (Lomas et al., 2017).

Therefore, the aim of this study was to evaluate the effectiveness of a Workplace Mindfulness Program (WMT) in improving health-related and performance-related outcomes. We wanted to investigate whether the WMT would improving burnout, psychological well-being, organizational and team climate and performance, using psychometrically valid self-report measures in a company setting. We hypothesized that the WMT would lead to greater improvements in mindfulness, stress and burnout symptoms, well-being, team and organizational climate and performance post-training compared to the pre-intervention period.

A secondary goal was to assess the acceptability and feasibility of the WMT in terms of meeting practice recommendations and completing the program.

To our knowledge, this is the first large-scale European study investigating the impact of a workplace mindfulness intervention in a multiple-company setting.

## Materials and Methods

### Design

This is a non-randomized pre–post evaluation with an additional assessment up to 1 month before start of the intervention using self-report questionnaires which were completed up to a month before (t0), at the start of (t1), and at the end of the training (t2) thus resulting in a pre-intervention period (t0–t1) and an intervention period (t1–t2). Initially, due to organizational and practical restraints, participants only filled out questionnaires before and after the training. At a later stage, participants were invited to complete questionnaires up to a month before the start of the training as well, or to contribute to the pre-intervention period only. Therefore, limited data are available for t0. For the current analyses, the four companies that included a pre-intervention period were selected.

IRB approval was granted by the University of Applied Sciences and Arts Coburg’s ethical committee.

### Procedure

Companies were enrolled by Kalapa Leadership Academy following presentations about the WMT at conferences or at their own companies. When a company agreed to participate, an information session was held for all potential participants within a company. Following that, employees could sign up for the WMT on a voluntary basis. The training was free of charge for employees and some companies specifically encouraged their leadership teams to take part. Companies completed the training between 2013 and 2016. Four companies were included in this study: A European skin care products company (company 1), a global automotive supplier (company 2), a European space research and technology organization (company 3), and a global pharmaceutical company (company 4).

To increase compliance, participants were offered an anonymized personal feedback report after the training and employers received an anonymized group feedback. Other than that, participants received no reimbursement for their participation in the research.

Self-report questionnaires were administered via an online survey battery. Additionally, participants were asked to document their practice frequency, type of practice and practice minutes via an online log book or excel sheet template. Data were collected anonymously and matched between different time points via a personal code (defined by participant). For privacy reasons, it was not possible to link this personal code to names, and therefore, it was not possible to follow up on missing responses.

### Intervention: Workplace Mindfulness Training

This WMT program named “WorkingMind” was developed on the conceptual basis of traditional mindfulness programs. It comprised of the philosophical grounding teaching of mindfulness (Sauer et al., 2011a) and psycho-education about the psychological and neuropsychological mechanism of action (Sauer et al., 2011b,c). Its formal structure resembles the traditional mindfulness programs, but it has been tailored to meet the needs and demands of employees (Sauer and Kohls, 2011; Kohls et al., 2013). Compared to other popular mindfulness programs like MBSR and MBCT, the WMT has a longer duration requiring participation in 2 day-long training days plus eight 2.5 h-long sessions. The WMT took place in a group setting with 12–25 participants per group. Additionally, attendees were provided with eight app-based audio recordings and encouraged to practice on their own. In response to the time restrictions of employees and in order to secure adherence to the program, participants were asked to practice mindfulness for at least 10 minutes daily. Participants learn a variety of formal and informal meditation practices including mindfulness meditation, walking meditation, pausing meditation, body scan and compassion meditation. Furthermore, participants are encouraged to practice mindfulness in everyday life (informal practices), for example mindful communication (listening, dialog), mindful team meetings (a minute silence before a group meeting), noticing positive experiences, mindful emailing and daily journaling (see Supplementary Tables 1, 2 for an overview of session themes, goals and methods). Moreover, the WMT’s comprehensive psychoeducational component includes information and discussions about the neurobiological response to stress and relaxation, the functioning of attentional networks, and the neurobiological basis for emotions and resilience.

The courses were delivered by two trainers who were experienced meditation practitioners with 10 or more years of personal practice, experience in handling group processes in a company setting as well as a good understanding of the relevant neuroscientific background.

### Outcome Measures

The Burnout Measure (BM; Pines et al., 1981) is a 21 item self-diagnostic measure of occupational burnout. Respondents rate their overall quality of life and their emotional, physical and mental exhaustion. A rating scale of (1) never to (7) always is used to score each item. Internal consistency is high ranging from α = 0.91 to α = 0.93 depending on the sample (Pines et al., 1981).

The Perceived Stress Questionnaire (PSQ; Fliege et al., 2005) is an instrument measuring different subcomponents of perceived stress. In addition to a total stress index, the following four sub-dimensions of stress are assessed: demands, tension, (lack of) joy and worries. The PSQ is rated on a 4-point Likert scale ranging from (1) ‘almost never’ to (4) ‘almost all the time.’ With the exception of the joy scale, a higher score indicates higher prevalence of stress. To calculate a total perceived stress score, joy scores are reversed and together with the remaining factors accumulated into a total stress index. This scale has shown good internal consistency in clinical populations as well as healthy adults (healthy adults *n* = 334, worries Cronbach’s α = 0.83, tension α = 0.77, joy α = 0.79, demands α = 0.77, overall score α = 0.86; Fliege et al., 2005).

The Freiburg Mindfulness Inventory (FMI-14; Walach et al., 2006; Kohls et al., 2009) is an instrument for measuring trait-mindfulness. The short version of the inventory comprises 14 items and is frequently used to evaluate changes in mindfulness with meditation novices. The FMI measures two aspects of mindfulness: mindful presence and mindful acceptance. Mindful presence is characterized by the ability to stay present or to return to the present moment when absent-minded. Mindful acceptance involves embracing negative experiences. Based on an item response analysis (IRT), results of a Rasch analysis conducted by Sauer et al. (2013) suggested to leave out the only negatively worded item 13 (“I am impatient with fellow human beings.”) in order to increase construct validity and internal consistency. Using 13-items, the total mindfulness scales and both subscales were statistically reliable at pretest (mindful presence: α = 0.83; mindful acceptance: α = 0.84). A higher mean score indicates higher trait-mindfulness for both sub-scales.

The Mindfulness Attention Awareness Scale (MAAS; Brown and Ryan, 2003) is a 15 item measure of the attentional aspect of mindfulness. All items are negatively phrased (Sample item: “I rush through activities without being really attentive to them.”). In this study, the answer choices ranged from (1) often to (4) never. All items were recoded so that a higher mean score indicates higher mindful attention. Internal consistency across various samples is good (α = 80 to α = 89; Brown and Ryan, 2003).

The WHO-Five well-being scale (WHO-5; WHO, 1998) is a scale to measure positive aspects of well-being. Respondents rate the experience of positive emotions over the course of the last weeks with five items. Participants report how often they felt in a joyful mood, relaxed, active, fresh and whether their day was filled with things of interest to them. In contrast to the literature where the items are scored from 0 (none of the time) to 5 (all of the time) (Topp et al., 2015), in our study the scale was rated on a scale ranging from (1) never to (7) always. Internal consistency in our sample was high (α = 0.85).

Landau Organization and Team Climate Inventory (LOTI; Müller, 2007) assesses various dimensions of organizational climate, team climate and personal performance. In this study, 7 scales comprised of a total of 44 items have been included. The LOTI is rated on a 7-point Likert scale ranging from (1) ‘never’ to (7) ‘always.’ Higher values indicate a more favorable organization or team climate with the exception of the pressure and stress scale. Team climate was measured with the scales (1) cooperation and collaboration, (2) leadership and organization and (3) decision-making and creativity. Organizational climate was assessed with the scales (1) appraisal and respect and (2) atmosphere and satisfaction. Personal performance was assessed with the scales (1) pressure and stress, and (2) productivity and concentration. In this study, internal consistency was good (appraisal and respect α = 0.70, atmosphere and satisfaction α = 0.78, cooperation and collaboration α = 0.77, decision-making and creativity α = 0.85, leadership and organization α = 0.87, pressure and stress α = 0.87, and productivity and concentration α = 0.85).

#### Program Feasibility and Satisfaction

Measures of program feasibility included (a) whether or not the training was completed; and (b) homework practice logs of the number of mindfulness practices and total practice minutes. The training was regarded as completed if an employee attended six or more out of the 10 sessions. The feasibility of practicing at home was assessed by how many employees met the guideline of practicing 10 minutes at least 5 days per week.

At the end of the WMI, participants were invited to complete the evaluation of the program as part of the t2 survey. Questions assessed satisfaction about the program in terms of employee’s perception of their overall satisfaction rating of the program.

### Description of the Sample

A total of 425 participants completed at least one of the surveys on the different time points. Because the study initially was a pre–post assessment only, most participants completed the surveys at t1 and t2 (*n* = 226, 53%). The pre-intervention period was added later, and resulted in 16 (4%) participants who completed the surveys at all time points (t0, t1, and t2) and 28 participants with completed surveys at t0 and t1 or at t0 and t2. A relatively large part of the subjects completed the survey at one time point only (t0, t1, or t2) (**Table 1**). This resulted in a total of 712 completed surveys. Most participants were from Company 1 (*n* = 126, 30%) and Company 2 (*n* = 213, 50%). Due to the set-up of the study, the number and percentage of participants of each company varied over the time points, with Company 3 contributing relatively more to t0, and Company 2 contributing more to t1 and t2 (**Table 2**).

**Table 1:** The number and percentage of subjects who completed surveys at start of the pre-intervention period (t0), end of pre-intervention/start of the intervention period (t1) and at the end of the intervention period (t2) or at a combination of these time points.

Time points	*N* (%)
**One time point**	
t0 only	23 (5)
t1 only	100 (24)
t2 only	32 (8)
**Two time points**	
t0 and t1	23 (5)
t0 and t2	5 (1)
t1 and t2	226 (53)
**Three time points**	
t0 and t1 and t2	16 (4)
**Total**	425

**Table 2:** Demographic and work characteristics of the study population in total and at start of the pre-intervention period (t0), end of the pre-intervention period/start of the intervention period (t1) and end of the intervention period (t2).

	All participants	Per time point		
		t0	t1	t2
**Total**	*N* = 425	*N* = 67	*N* = 366	*N* = 279
**Gender, *n* (%)**	*N* = 356	*N* = 51	*N* = 340	*N* = 233
Male	212 (60)	20 (39)	205 (60)	134 (58)
Female	144 (40)	31 (61)	135 (40)	99 (43)
**Age (mean, SD)**	*N* = 360	*N* = 62	*N* = 335	*N* = 235
	43.9 (8)	46.5 (8)	43.6 (8)	43.8 (8)
**Marital state, *n* (%)**	*N* = 366	*N* = 62	*N* = 342	*N* = 241
Single or unmarried	87 (24)	12 (19)	83 (24)	57 (24)
Married	250 (68)	38 (61)	235 (69)	171 (71)
Divorced	29 (8)	12 (19)	24 (7)	13 (5)
**Children, *n* (%)**	*N* = 365	*N* = 61	*N* = 342	*N* = 239
No	112 (31)	15 (25)	108 (32)	76 (32)
Yes	253 (69)	46 (75)	234 (68)	163 (68)
**Living situation, *n* (%)**	*N* = 353	*N* = 49	*N* = 337	*N* = 232
Living alone	51 (14)	15 (31)	46 (14)	28 (12)
Living with partner or spouse	297 (84)	34 (69)	286 (85)	202 (87)
Living in shared flat	4 (1)	0 (0)	4 (1)	1 (0)
Living with parents	1 (0)	0 (0)	1 (0)	1 (0)
**Education, *n* (%)**	*N* = 367	*N* = 63	*N* = 340	*N* = 239
Vocational training	46 (11)	7 (11)	42 (12)	32 (13)
University degree (BA, MA)	262 (62)	38 (60)	243 (71)	171 (72)
Ph.D.	55 (13)	15 (24	53 (16)	34 (14)
Other	4 (1)	3 (5)	2 (1)	2 (1)
**Company, *n* (%)**	*N* = 425	*N* = 67	*N* = 366	*N* = 279
Company 1	126 (30)	18 (27)	108 (30)	94 (34)
Company 2	213 (50)	12 (18)	192 (52)	141 (51)
Company 3	35 (8)	26 (39)	22 (6)	22 (8)
Company 4	51 (12)	11 (16)	44 (12)	22 (8)
**Leadership role, *n* (%)**	*N* = 364	*N* = 61	*N* = 339	*N* = 237
Employee or other	204 (56)	38 (62)	188 (56)	135 (57)
Leader	160 (44)	23 (38)	151 (55)	102 (43)
**Working hours (mean, SD)**	*N* = 360 **(t1)**	*N* = 67	*N* = 360	*N* = 277
	46,3 (10)	45.5 (9)	46.3 (10)	46.2 (11)
**Working load, *n* (%)**	*N* = 362 **(t1)**	*N* = 67	*N* = 362	*N* = 279
Very high	101 (28)	16 (24)	101 (28)	73 (26)
Rather high	240 (66)	42 (63)	240 (66)	181 (65)
Moderate	21 (6)	9 (13)	21 (6)	24 (9)
Low	0 (0)	0 (0)	0 (0)	1 (0)

### Statistical Analysis

Linear mixed model analysis (SPSS version 22) was used to estimate mean scores on the different time points and the mean changes within the pre-intervention (t0 and t1) and intervention period (t1 and t2).

Because two comparisons were tested (t0 and t1, and t1 and t2), Bonferroni correction for multiple testing was applied by multiplying *p*-values by two for the pre–post assessments within the intervention and pre-intervention period. The mean difference between the pre-intervention and intervention periods (intervention effect) was estimated by subtracting the effect in the pre-intervention period from the effect in the intervention period.

Linear mixed models take into account the nesting of repeated measurements within individuals. One advantage of this modeling is that all data points can be included in the analyses, independent of completeness of the data, thereby avoiding further selection of the data. The model assumes that the participants consist of an a-select sample of a background population that we are interested in, and estimates mean outcome scores for each time point based on the available data (Brown and Prescott, 2015).

An additional analysis was performed among a subsample of participants who had complete data on t0, t1, and t2 (*n* = 16) to check whether the results supported the findings in the whole sample (Supplementary Table 3).

Furthermore, an analysis was conducted to evaluate whether differences in socio-demographic and work characteristics between time points may have confounded the results. The adjusted effects were similar to the effects of the initial analyses (Supplementary Table 4), and because the data were more complete without adjusting for covariates, the raw differences were regarded as the main result.

*P*-values of <0.05 were considered statistically significant and *p*-values of <0.05, <0.01, and <0.001 were presented. Cohen’s *d* type of effect sizes were calculated based on the model estimated mean difference between the effects in the intervention and the pre-intervention period divided by the square root of the variance between participants (intercept). These effect sizes are generally interpreted as small (0.2–0.5), medium (0.5–0.8), and large (>0.8) (Cohen, 1988).

## Results

### Demographics and Work Characteristics

The demographic and work characteristics are presented in **Table 2**. Participants were on average 43.9 years of age (*SD* = 8.0). The majority of participants were male (60%), married (68%), lived with their partner or spouse (84%) and had children (69%). Most of the participants had a university degree (62%). In total, 80% of the participants were from Company 1 (30%) and Company 2 (50%). The average number of working hours on t1 was 46 (*SD* = 10), and most participants experienced their workload as very high (*n* = 101, 28%) or rather high (*n* = 240, 66%).

### Burnout and Perceived Stress

Significantly greater reductions in burnout and perceived stress were reported during the training than in the pre-intervention period. Similar results were found for the Perceived Stress Questionnaire (PSQ) subscales, with greatest improvements in PSQ tension. These were all medium effects (**Table 3**).

**Table 3:** Mean scores (SE) at the time points, differences (SE) between the time points and periods, and effect sizes for burnout and perceived stress.

	Mean	Mean	Mean	Mean	Mean	Mean difference	Cohen’s *d*
	(*SE*)^1^	(*SE*)^1^	(*SE*)^1^	difference	difference	between periods	type of effect
	t0	t1	t2	t0 to t1 (*SE*)^2^	t1 to t2 (*SE*)^2^	(*SE*)^3^	size^4^
**Burnout**	*n* = 65	*n* = 357	*n* = 276				
Burnout Measure	3.2 (0.07)	3.2 (0.04)	2.9 (0.04)	0.0 (0.07)	–0.3 (0.03)^∗∗∗^	–0.3 (0.08)^∗∗∗^	–0.5
**Perceived stress**	*n* = 67	*n* = 363	*n* = 279				
PSQ Total (1–4)	2.4 (0.04)	2.4 (0.02)	2.2 (0.02)	0.0 (0.04)	–0.2 (0.02)^∗∗∗^	–0.2 (0.04)^∗∗∗^	–0.6
PSQ Demands (1–4)	2.8 (0.06)	2.8 (0.03)	2.7 (0.03)	0.1 (0.06)	–0.2 (0.03)^∗∗∗^	–0.2 (0.07)^∗∗^	–0.5
PSQ Tension (1–4)	2.5 (0.05)	2.6 (0.03)	2.3 (0.03)	0.1 (0.05)	–0.3 (0.03)^∗∗∗^	–0.3 (0.06)^∗∗∗^	–0.8
PSQ Joy (1–4)	2.6 (0.05)	2.6 (0.03)	2.8 (0.03)	0.1 (0.05)	0.2 (0.03)^∗∗∗^	0.1 (0.06)^∗^	0.3
PSQ Worry (1–4)	2.0 (0.05)	2.0 (0.03)	1.8 (0.03)	0.0 (0.05)	–0.2 (0.02)^∗∗∗^	–0.2 (0.06)^∗∗∗^	–0.5

*^*1*^Means were estimated by the model.*

*^*2*^*P*-values multiplied by 2 to adjust for multiple testing (Bonferroni).*

*^*3*^The mean difference between periods was calculated by subtracting the effect in the pre-intervention period from the effect in the intervention period.*

*^*4*^Calculated based on the model estimated mean difference between effect is intervention and pre-intervention period divided by the square root of the variance between participants (intercept).*

*^∗^*P* < 0.05, ^∗∗^*P* < 0.01, ^∗∗∗^*P* < 0.001.*

### Psychological Well-Being: Mindfulness and Well-Being

Mindfulness as measured by two scales improved significantly during the training. When compared to the pre-intervention period, both the FMI score and its subscales presence and acceptance significantly increased in the intervention period, with FMI Presence contributing more to the increase than FMI Acceptance (**Table 4**). The increased mindfulness was also mirrored in the MAAS scores, which increased significantly greater during the intervention period compared to the pre-intervention period. The effect sizes for the mindfulness measures were 0.8 or higher indicating large effect sizes. Medium training effects were observed for the well-being measure (**Table 4**).

**Table 4:** Mean scores (SE) at the time points, differences (SE) between the time points and periods, and effect sizes for mindfulness and well-being.

	Mean	Mean	Mean	Mean	Mean	Mean difference	Cohen’s *d*
	(*SE*)^1^	(*SE*)^1^	(*SE*)^1^	difference	difference	between periods	type of effect
	t0	t1	t2	t0 to t1 (*SE*)^2^	t1 to t2 (*SE*)^2^	(*SE*)^3^	size^4^
**Mindfulness**	*n* = 67	*n* = 366	*n* = 279				
FMI Total (1–4)	3.0 (0.05)	2.8 (0.03)	3.1 (0.03)	–0.1 (0.05)^∗∗^	0.3 (0.02)^∗∗∗^	0.4 (0.06)^∗∗∗^	1.0
FMI Presence (1–4)	3.0 (0.06)	2.8 (0.03)	3.2 (0.03)	–0.2 (0.06)^∗∗^	0.4 (0.03)^∗∗∗^	0.6 (0.07)^∗∗∗^	1.3
FMI Acceptance (1–4)	3.0 (0.05)	2.9 (0.03)	3.1 (0.03)	–0.1 (0.05)^∗^	0.2 (0.03)^∗∗∗^	0.3 (0.06)^∗∗∗^	0.8
	*n* = 67	*n* = 366	*n* = 279				
MAAS (1–4)	2.4 (0.05)	2.4 (0.02)	2.6 (0.03)	–0.05 (0.05)	0.2 (0.03)^∗∗∗^	0.3 (0.06)^∗∗∗^	0.8
**Well-being**	*n* = 67	*n* = 362	*n* = 279				
WHO-5 Well-being (1–7)	4.4 (0.09)	4.4 (0.05)	4.8 (0.05)	–0.02 (0.1)	0.4 (0.05)^∗∗∗^	0.4 (0.1)^∗∗∗^	0.6

*^*1*^Means were estimated by the model.*

*^*2*^*P*-values multiplied by 2 to adjust for multiple testing (Bonferroni).*

*^*3*^The mean difference between periods was calculated by subtracting the effect in the pre-intervention period from the effect in the intervention period.*

*^*4*^Calculated based on the model estimated mean difference between effect is intervention and pre-intervention period divided by the square root of the variance between participants (intercept).*

*^∗^*P* < 0.05, ^∗∗^*P* < 0.01, ^∗∗∗^*P* < 0.001.*

### Organizational Climate, Team Climate, and Personal Performance

Most scales measuring team and organizational climate as well as personal performance improved significantly after the training when compared to the pre-intervention period.

In regards to team climate, significant increases were observed for the subscales LOTI cooperation and LOTI decision-making. The participants also judged their leaders more favorable after the intervention period, compared to the pre-intervention period. However, the difference between the two periods was not statistically significant. Significantly greater increases were reported for organizational climate (subscales LOTI respect and LOTI atmosphere) during the intervention compared to the pre-intervention period. Moreover, personal performance significantly improved after the intervention, as indicated by an increase in self-reported productivity and a decrease in self-reported stress after the training when compared to the pre-intervention period. Effect sizes were generally small to medium (**Table 5**).

**Table 5:** Mean scores (SE) at the time points, differences (SE) between the time points and periods, and effect sizes for team climate, organizational climate and personal performance.

	Mean (*SE*)^1^ t0	Mean (*SE*)^1^ t1	Mean (*SE*)^1^ t2	Mean difference t0 to t1 (*SE*)^2^	Mean difference t1 to t2 (*SE*)^2^	Mean difference between periods (*SE*)^3^	Cohen’s *d* type of effect size^4^
**Team climate**	*n* = 67	*n* = 366	*n* = 279				
Loti Cooperation (1–7)	5.6 (0.06)	5.4 (0.03)	5.6 (0.03)	–0.2 (0.03)^∗∗^	0.2 (0.03)^∗∗∗^	0.3 (0.08)^∗∗∗^	0.7
Loti Leadership (1–7)	5.5 (0.08)	5.5 (0.04)	5.6 (0.05)	0.0 (0.08)	0.2 (0.04)^∗∗∗^	0.1 (0.09)	0.2
Loti Decision (1–7)	5.2 (0.07)	5.2 (0.04)	5.4 (0.04)	0.0 (0.07)	0.2 (0.04)^∗∗∗^	0.2 (0.08)^∗∗^	0.4
**Organizational climate**							
Loti Respect (1–7)	5.4 (0.07)	5.4 (0.04)	5.7 (0.04)	–0.0 (0.08)	0.2 (0.04)^∗∗∗^	0.3 (0.09)^∗∗^	0.4
Loti Atmosphere (1–7)	5.6 (0.07)	5.6 (0.04)	5.8 (0.04)	–0.0 (0.07)	0.2 (0.03)^∗∗∗^	0.2 (0.08)^∗∗^	0.4
**Personal performance**							
Loti Productivity (1–7)	4.8 (0.09)	4.7 (0.05)	5.1 (0.05)	–0.1 (0.09)	0.4 (0.05)^∗∗∗^	0.5 (0.11)^∗∗∗^	0.8
Loti Stress (1–7)	3.7 (0.08)	3.7 (0.04)	3.3 (0.05)	0.0 (0.08)	–0.4 (0.04)^∗∗∗^	–0.4 (0.09)^∗∗∗^	–0.6

*^*1*^Means were estimated by the model.*

*^*2*^*P*-values multiplied by 2 to adjust for multiple testing (Bonferroni).*

*^*3*^The mean difference between periods was calculated by subtracting the effect in the pre-intervention period from the effect in the intervention period.*

*^*4*^Calculated based on the model estimated mean difference between effect is intervention and pre-intervention period divided by the square root of the variance between participants (intercept).*

*^∗^*P* < 0.05, ^∗∗^*P* < 0.01, ^∗∗∗^*P* < 0.001.*

### Additional Analysis Among 16 Participants With Complete Data

When the analyses were restricted to the 16 participants with complete data on all three time points, the mean differences between the intervention and pre-intervention period were similar in direction and order of magnitude, or even increased compared to the initial analyses. However, statistical power was reduced, as can be expected with this small number of subjects, resulting in less significant findings and/or lower levels of statistical significance (Supplementary Table 3).

### Additional Analysis Including Covariates

Demographics and work characteristics were generally comparable between t1 and t2, but some differed between t0 and the other time points. This was most pronounced for the demographic variables gender (more females at t0), children (more had children at t0), living situation (more living alone at t0), and leadership position (less leaders at t0). Moreover, the four companies contributed differently to each time point (**Table 2**). Therefore, these variables were included as covariates in the model. The adjusted effects were comparable to the effects in the initial analyses (Supplementary Table 4).

### Program Feasibility and Satisfaction

Weekly attendance was reported by 279 attendees, however, the data of 10 respondents was either incomplete or faulty (e.g., reporting a higher number of missed sessions than actual training sessions) resulting in a total of 269 respondents included in the analysis.

Out of the 269 respondents that recorded their training participation, 265 (98.5%) had attended six or more out of the 10 sessions and therefore met our requirement for completion of the program. Most participants (253, 94.1%) missed three or fewer course sessions (38 missed no session, 61 missed one session, 106 missed two sessions, and 48 missed three sessions). Over the 10 weeks of the training, participants practiced meditation on average 4.5 times per week (*SD* = 2.9) and 45.7 minutes per week (*SD* = 343).

Practice logs were returned from 198 attendees. Of these, 62 (31.3%) engaged in less than 5 minutes of mindfulness practice per day and 64 (32.3%) practiced between 5 and 10 minutes. Thirty-nine (19.7%) participants practiced between 10 and 15 minutes and 31 (15.7%) more than 15 minutes daily. In summary, 35.4% of participants practiced for 10 or more minutes per day.

A total of 279 participants gave feedback on their satisfaction with the program. Most participants reported that the program had been “very valuable for me” (255, 91.7%) and “for the company as a whole” (222, 79.6%) indicating that the program acceptability was high.

## Discussion

As far as we are aware, this is the first large scale study evaluating the effects of a mindfulness program in the workplace on burnout, psychological well-being and performance across multiple corporations.

Our preliminary data suggest significant reductions in self-reported burnout and perceived stress, and improvements in mindfulness and perceived well-being over the intervention period compared to the pre-intervention period. Moreover, significant improvements in key organizational outcomes including personal performance and productivity, cooperation within teams and organizational climate were observed over the intervention period but not the pre-intervention period.

Feasibility of the program was high with 98.5% of respondents attending six or more out of 10 sessions. The majority of participants who filled in the log books engaged in daily meditation practice but only 35.4% met the target of practicing 10 or more minutes daily. Satisfaction with the program was very high amongst participants indicating that they enjoyed participation and benefitted from it.

Due to various limitations in our study design, the results should be interpreted with care. An obvious limitation of the study is the absence of a randomized control group, relatively small amount data at t0, and incompleteness of the data. Clearly, our data do not comply with the scientific standards to evaluate interventions (Schulz et al., 2010). This can be attributed to the field study nature of data collection and organizational restraints as well as practical limitations in this setting. It proved difficult to convince companies of the importance of a control group or a pre-intervention period. The small number of participants that contributed to t0 and therefore to a pre-intervention period was the best feasible option in this setting. Despite this, it is considered a strength of the study that at least some pre-intervention period data were included, which is often not done in company settings.

By using linear mixed model analysis, the mean scores were estimated for all time points based on the available data. The strength of this analysis is that no data had to be removed and a further possible selection of the data was avoided. The model assumes that participants with missing data are comparable to individuals with complete data in regards to intervention effects. As it was not known which and how many participants were invited to the different periods and time points, it was not possible to define non-responders and consequently we could not perform a non-responder analysis. However, the model assumption was corroborated by the fact that a subgroup analysis restricted to the 16 participants who completed the surveys at all three time points yielded effects comparable to those observed in the main analysis. As can be expected in a non-randomized study, some differences in socio-demographics and work characteristics were observed between participants at t0 compared to those at t1 and t2. However, the additional analysis among participants contributing to three time points indicate that results are unlikely to be caused by differences in participants between the three time points (see Supplementary Table 3). An additional analysis was conducted in which these covariates were included in the model, further strengthening the assumption that the differences observed in outcomes between intervention and pre-intervention period were not caused by differences in socio-demographic variables or work characteristics between the time points (see Supplementary Table 4).

The validity of the pre-intervention period data was further supported by the comparability of the mean scores on t0 and t1 for all outcomes, which would be expected for a pre-intervention period. Only for the mindfulness scales, a slight decrease was observed in the pre-intervention period. Participants may become more sensitive to their not being mindful in anticipation of starting with a MT, as is also reported for mindfulness trainers (Sauer et al., 2014).

Although the limited pre-intervention period data and the non-randomized design warrant careful interpretation (Shadish et al., 2002), the above additional analyses support the interpretation that differences found between intervention and pre-intervention period may be attributed to the training. However, on the basis of this study no conclusions can be drawn on whether the results are due to the mindfulness component of the training specifically, or to non-specific effects of study participation, or due to time effects. Other shortcomings are the self reported outcomes which may have resulted in social desirable responses, and the lack of a follow up period so that it is not known whether the effects would last in time. Due to these limitations in the design, the associations found may not be replicated when a randomized controlled trial would be applied.

Our preliminary findings are in line with previous investigations of the impact of MTs on reducing stress (Khoury et al., 2013) and of workplace mindfulness interventions on employee well-being, stress and burnout (Good et al., 2016; Lomas et al., 2017). The amelioration of personal well-being and performance might be attributed to improvements of functional aspects that are encompassed by the state of mindfulness, namely attention regulation and emotion regulation (Hölzel et al., 2011). While many studies have shown the link between mindfulness practices and cognitive performance in a lab setting (Systematic Review: Chiesa et al., 2011), few have specifically investigated this in a workplace setting.

Other than previous workplace studies, this study included both individual *and* workplace outcomes, such as measures of individual well-being, performance and relational aspects such as cooperation, group atmosphere, and respect. A common critique of mindfulness in business settings is that it might be employed to increase productivity at the expense of investing the improvement of working environments. Our study indeed indicated a possible increase in self-rated employee productivity. At the same time, however, not only individual well-being, but also self-assessed quality of organizational climate may improve, in terms of better cooperation, better working atmosphere and more respect among team members. This suggests that not only intrapersonal variables such as self-reported functionality may improve but also interpersonal parameters such as team-climate and social welfare.

Future field studies should aim at collecting more complete data and more control data. Preferably, more rigorous designs should be employed, such as Randomized Controlled Trials (RCTs) or designs that are adaptable to the needs of the workplace (e.g., stepped-wedge design; Copas et al., 2015; Hemming et al., 2015).

Furthermore, effects on leadership as well as the mechanisms of change in workplace outcomes will need to be investigated carefully. It might be interesting to include additional objective measures to assess the impact of mindfulness interventions on performance, such as absenteeism and presenteeism, and team collaboration, such as team member interviews or a group task. Future research needs to investigate the long-term sustainability of the program benefits for both the employees and the organizations. It is recommended to collect longer term follow-up data up to 6 or 12 months after the training to assess the stability of the post intervention effects. Research should also turn its attention to assessing different modes of delivery of training by comparing the effectiveness of more cost- and time-effective methods of delivery (webinar, app-based trainings) to traditional live-training formats.

This preliminary field study indicated that the WMT was associated with reductions in burnout and perceived stress and improvements in mindfulness, perceived well-being, and several aspects of team and organizational climate and personal performance. Further studies, preferably using randomized controlled study designs are needed to evaluate whether the associations found can be attributed to the WMT. Interventions which enhance workplace well-being and performance are not only valuable for an employee’s well-being and reduction of chronic stress and mental health issues, but would also have considerable positive benefits for organizations and the society as whole.

## Ethics Statement

The study was approved by the Ethics Committees of the University of Coburg. All participants were informed about the contents of the research, participation was voluntary and they had the right to withdraw at any time. Data were collected anonymously, it was not possible to relate the data to any personal information.

## Author Contributions

CT, NK, MW, and PF designed or planned the study and collected the data. WK, SR, and RD designed and conducted the statistical analyses. WK and SR drafted the manuscript. All authors interpreted the data, provided substantive suggestions for revisions and critically reviewed subsequent versions of the manuscript, and reviewed and approved the final version of the manuscript.

## Conflict of Interest Statement

The research was funded by Kalapa Leadership Academy, CT is the owner of Kalapa Leadership Academy, and PF is employed by Kalapa Leadership Academy. NK, WK, and SR were hired by Kalapa Leadership Academy to conduct the work. The other authors declare that the research was conducted in the absence of any commercial or financial relationships that could be construed as a potential conflict of interest.
