# Supplementary material for: A Workplace Mindfulness Intervention May Be Associated With Improved Psychological Well-Being and Productivity. A Preliminary Field Study in a Company Setting
Source: Front Psychol. 2018 Feb 28;9:195. doi: 10.3389/fpsyg.2018.00195 (PMC5836057; doi:10.3389/fpsyg.2018.00195)
Supplement: Supplementary file 1 [file Table_1.docx]

Supplementary Material

**A Workplace Mindfulness Intervention May Be Associated with Improved Psychological Well-Being and Organizational Outcomes. A Preliminary Field Study in a Company Setting.**

Wendy Kersemaekers^1*†^, Silke Rupprecht^1†^, Marc Wittmann^2,3^, Chris Tamdjidi^4^, Pia Falke^4^, Rogier Donders^5^, Anne Speckens^1^, Niko Kohls^6^

*1 Radboudumc Center for Mindfulness, Department of Psychiatry, Radboud University Medical Center, Nijmegen, The Netherlands, 2 Institute for Areas of Psychology and Mental Health, Freiburg, Germany, 3 Institute of Medical Psychology, Ludwig-Maximilian University of Munich, Munich, Germany, 4 Kalapa Leadership Academy, Cologne, Germany, 5 Department for Health Evidence, Radboud University Medical Center, Nijmegen, Netherlands, 6 Division of Integrative Health Promotion, University of Applied* *Sciences and Arts, Coburg, Germany*

*** Correspondence:** *Wendy.kersemaekers@radboudumc.nl*

***^†^*** *These authors contributed equally to this work.*

# Supplementary Tables

**
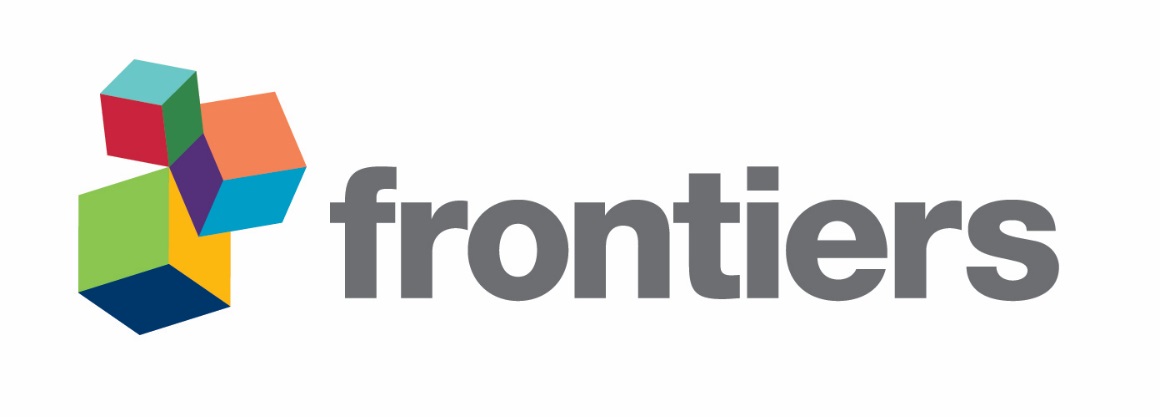
**

**Supplementary Table 1.** **Overview of modules of the Workplace Mindfulness Training**

| Module | Goals | Methods |
| --- | --- | --- |
| Deepening day | Introduction to neuroscience. Brain science and neuroplasticity. Getting to know mindfulness.  Mindfulness practice and discussions | Mindfulness meditation  Walking meditation  Reflection and journaling |
| Module 1: Attention and focus | Neuroscience of attention. Understanding and experiencing how multi-tasking negatively impacts attention and wellbeing | Mindfulness practice  Multi-tasking exercise  Mindful e-Mailing |
| Module 2: Emotions | Neuroscience of emotions. Becoming curious about emotions and developing self-compassion. | Body scan  4 step Method of working with emotions |
| Module 3: Happiness | Neuroscience of happiness. Learning how to cultivate happiness and how it impacts engagement and performance | Mindful appreciation  Aimless wandering |
| Module 4: Time | Neuroscience of time perception. Moving from time management to managing our attention, Learning about flow experiences | Time sensation exercise  Reflection on time perception  One minute pause |
| Module 5: Review and transitions | Deepening previous learning and strengthening practice. Mindful Rituals | Mindfulness meditation  Compassion meditation |
| Module 6: Communication | Social neuroscience and empathy. Extending mindfulness to communication with others | Mindful listening  Deepening in mindful dialogs |
| Module 7: Collaboration and trust | Social neuroscience and trust in team work. Recognising that human relationships underpin all collaboration | Mindful dialogues  Appreciative reflection  Mindful feedback  Mindful meetings |
| Module 8: Self-management and leadership | Understanding the role mindfulness plays in leading our own life and in cultivating authenticity in leadership | Space-awareness exercises  Reflection on decision making  Leadership exercise |
| Final Day | Reflection and commitment for future mindfulness practice | Walking through the module stations  Open Space – gathering of topics  World Café – Dialogue  Final presentation of topics |
