# Supplementary material for: A Workplace Mindfulness Intervention May Be Associated With Improved Psychological Well-Being and Productivity. A Preliminary Field Study in a Company Setting
Source: Front Psychol. 2018 Feb 28;9:195. doi: 10.3389/fpsyg.2018.00195 (PMC5836057; doi:10.3389/fpsyg.2018.00195)
Supplement: Supplementary file 2 [file Table_2.docx]

Supplementary Material

**A Workplace Mindfulness Intervention May Be Associated with Improved Psychological Well-Being and Organizational Outcomes. A Preliminary Field Study in a Company Setting.**

Wendy Kersemaekers^1*†^, Silke Rupprecht^1†^, Marc Wittmann^2,3^, Chris Tamdjidi^4^, Pia Falke^4^, Rogier Donders^5^, Anne Speckens^1^, Niko Kohls^6^

*1 Radboudumc Center for Mindfulness, Department of Psychiatry, Radboud University Medical Center, Nijmegen, The Netherlands, 2 Institute for Areas of Psychology and Mental Health, Freiburg, Germany, 3 Institute of Medical Psychology, Ludwig-Maximilian University of Munich, Munich, Germany, 4 Kalapa Leadership Academy, Cologne, Germany, 5 Department for Health Evidence, Radboud University Medical Center, Nijmegen, Netherlands, 6 Division of Integrative Health Promotion, University of Applied* *Sciences and Arts, Coburg, Germany*

*** Correspondence:** *Wendy.kersemaekers@radboudumc.nl*

***^†^*** *These authors contributed equally to this work.*

# Supplementary Tables

**
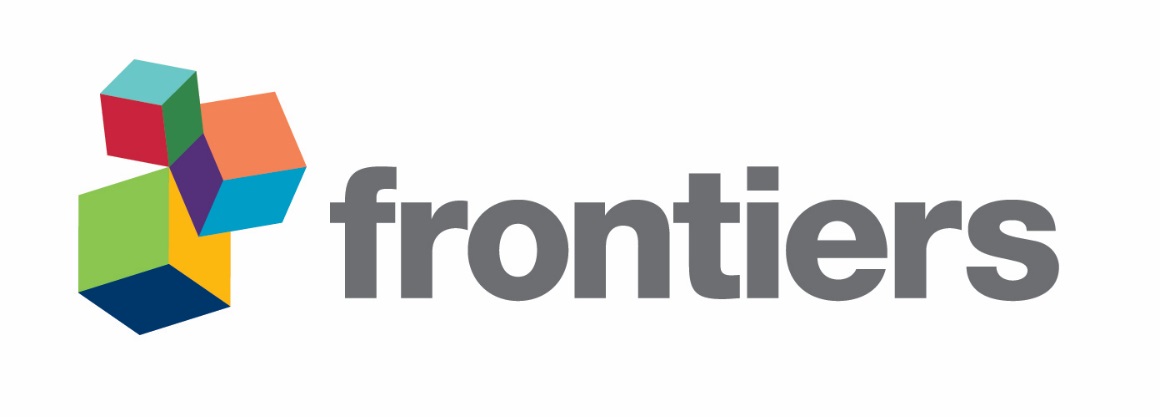
**

**Supplementary Table 2.** **Overview of typical Practice Session**

- Practice: Meditation practice of about 10 minutes, e.g. mindfulness meditation
- Check-In: Attendees share their experiences regarding home practices in the last week and their current state of being (first in small groups of 2-3, then in the whole group)
- Introduction to theme: Trainers introduce topic of this session backed by research findings
- Exercise: Exercise or practice session is linked to topic
- Group discussion: In small groups or the whole group the presented topic is discussed and applied to personal and work life
- Home Practice and handouts: Participants receive invitation for further practice and handouts if they want to deepen their understanding of the theme.
- Reflection and journaling
- Potentially further mindfulness practice specifically related to content (for example practice mindful listening)
- Check-out
