# Supplementary material for: A Workplace Mindfulness Intervention May Be Associated With Improved Psychological Well-Being and Productivity. A Preliminary Field Study in a Company Setting
Source: Front Psychol. 2018 Feb 28;9:195. doi: 10.3389/fpsyg.2018.00195 (PMC5836057; doi:10.3389/fpsyg.2018.00195)
Supplement: Supplementary file 3 [file Table_3.docx]

Supplementary Material

**A Workplace Mindfulness Intervention May Be Associated with Improved Psychological Well-Being and Organizational Outcomes. A Preliminary Field Study in a Company Setting.**

Wendy Kersemaekers^1*†^, Silke Rupprecht^1†^, Marc Wittmann^2,3^, Chris Tamdjidi^4^, Pia Falke^4^, Rogier Donders^5^, Anne Speckens^1^, Niko Kohls^6^

*1 Radboudumc Center for Mindfulness, Department of Psychiatry, Radboud University Medical Center, Nijmegen, The Netherlands, 2 Institute for Areas of Psychology and Mental Health, Freiburg, Germany, 3 Institute of Medical Psychology, Ludwig-Maximilian University of Munich, Munich, Germany, 4 Kalapa Leadership Academy, Cologne, Germany, 5 Department for Health Evidence, Radboud University Medical Center, Nijmegen, Netherlands, 6 Division of Integrative Health Promotion, University of Applied* *Sciences and Arts, Coburg, Germany*

*** Correspondence:** *Wendy.kersemaekers@radboudumc.nl*

***^†^*** *These authors contributed equally to this work.*

# Supplementary Tables

**
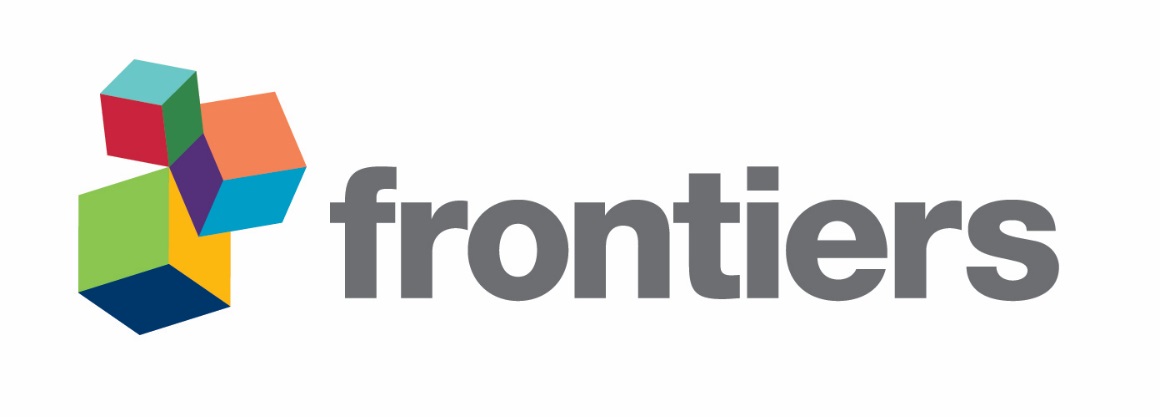
**

**Supplementary Table 3. Mean scores (SE) at the timepoints and differences (SE) between the pre-intervention and intervention periods for participants who completed the surveys at all three time points compared to the results of the total sample.**

|  | Mean (SE)^1^  T0 | Mean (SE)^1^  T1 | Mean (SE)^1^  T2 | Mean difference between periods (SE)  subsample^2^ | Mean difference between periods (SE) total sample^2^ |
| --- | --- | --- | --- | --- | --- |
| **Total n** | 16 | 16 | 16 |  |  |
| **Burnout** |  |  |  |  |  |
| Burnout Measure Total (1-7) | 3.3 (0.1) | 3.2 (0.2) | 2.9 (0.2) | -0.2 (0.2) | -0.3 (0.08)*** |
|  |  |  |  |  |  |
| **Perceived stress** |  |  |  |  |  |
| PSQ Total (1-4) | 2.5 (0.1) | 2.5 (0.1) | 2.2 (0.1) | -0.2 (0.1)* | -0.2 (0.04)*** |
| PSQ Demands (1-4) | 2.7 (0.1) | 2.7 (0.1) | 2.6 (0.1) | -0.1 (0.2) | -0.2 (0.07)** |
| PSQ Tension (1-4) | 2.5 (0.2) | 2.5 (0.2) | 2.2 (0.2) | -0.4 (0.1)** | -0.3 (0.06)*** |
| PSQ Joy (1-4) | 2.3 (0.1) | 2.5 (0.1) | 2.7 (0.1) | -0.0 (0.1) | 0.1 (0.06)* |
| PSQ Worry (1-4) | 2.1 (0.2) | 2.2 (0.2) | 1.9 (0.2) | -0.4 (0.2)* | -0.2 (0.06)*** |
|  |  |  |  |  |  |
| **Mindfulness** |  |  |  |  |  |
| FMI Total (1-4) | 2.9 (0.1) | 2.7 (0.1) | 3.2 (0.1) | 0.6 (0.1)*** | 0.4 (0.06)*** |
| FMI Presence (1-4) | 2.9 (0.2) | 2.8 (0.2) | 3.2 (0.2) | 0.6 (0.2)* | 0.6 (0.07)*** |
| FMI Acceptance (1-4) | 2.8 (0.1) | 2.7 (0.1) | 3.1 (0.1) | 0.6 (0.2)*** | 0.3 (0.06)*** |
|  |  |  |  |  |  |
| MAAS (1-4) | 2.3 (0.1) | 2.2 (0.1) | 2.6 (0.1) | 0.4 (0.2)* | 0.3 (0.06)*** |
|  |  |  |  |  |  |
| **Well-being** |  |  |  |  |  |
| Well-being (1-7) | 4.2 (0.2) | 4.3 (0.2) | 4.7 (0.2) | 0.3 (0.3) | 0.4 (0.1)*** |
|  |  |  |  |  |  |
| **Organisational, team Climate, Personal performance** |  |  |  |  |  |
| Loti Cooperation (1-7) | 5.4 (0.2) | 5.1 (0.2) | 5.5 (0.2) | 0.6 (0.3)* | 0.3 (0.08)*** |
| Loti Leadership (1-7) | 4.9 (0.2) | 4.9 (0.2) | 5.3 (0.2) | 0.5 (0.2) | 0.1 (0.09) |
| Loti Decision (1-7) | 4.8 (0.2) | 4.7 (0.2) | 5.1 (0.2) | 0.5 (0.2)* | 0.2 (0.08)** |
|  |  |  |  |  |  |
| Loti Respect (1-7) | 5.2 (0.2) | 5.1 (0.2) | 5.5 (0.2) | 0.5 (0.3) | 0.3 (0.09)** |
| Loti Atmosphere (1-7) | 5.5 (0.2) | 5.5 (0.2) | 5.8 (0.2) | 0.2 (0.2) | 0.2 (0.08)** |
|  |  |  |  |  |  |
| Loti Productivity (1-7) | 4.5 (0.2) | 4.5 (0.2) | 5.0 (0.2) | 0.6 (0.3) | 0.5 (0.11)*** |
| Loti Stress (1-7) | 4.0 (0.2) | 4.2 (0.2) | 3.6 (0.2) | -0.8 (0.2)*** | -0.4 (0.09)*** |

^*^ P <0.05

^**^ P <0.01

^***^ P <0.001
