# Supplementary material for: A Workplace Mindfulness Intervention May Be Associated With Improved Psychological Well-Being and Productivity. A Preliminary Field Study in a Company Setting
Source: Front Psychol. 2018 Feb 28;9:195. doi: 10.3389/fpsyg.2018.00195 (PMC5836057; doi:10.3389/fpsyg.2018.00195)
Supplement: Supplementary file 4 [file Table_4.docx]

Supplementary Material

**A Workplace Mindfulness Intervention May Be Associated with Improved Psychological Well-Being and Organizational Outcomes. A Preliminary Field Study in a Company Setting.**

Wendy Kersemaekers^1*†^, Silke Rupprecht^1†^, Marc Wittmann^2,3^, Chris Tamdjidi^4^, Pia Falke^4^, Rogier Donders^5^, Anne Speckens^1^, Niko Kohls^6^

*1 Radboudumc Center for Mindfulness, Department of Psychiatry, Radboud University Medical Center, Nijmegen, The Netherlands, 2 Institute for Areas of Psychology and Mental Health, Freiburg, Germany, 3 Institute of Medical Psychology, Ludwig-Maximilian University of Munich, Munich, Germany, 4 Kalapa Leadership Academy, Cologne, Germany, 5 Department for Health Evidence, Radboud University Medical Center, Nijmegen, Netherlands, 6 Division of Integrative Health Promotion, University of Applied* *Sciences and Arts, Coburg, Germany*

*** Correspondence:** *Wendy.kersemaekers@radboudumc.nl*

***^†^*** *These authors contributed equally to this work.*

# Supplementary Tables

**
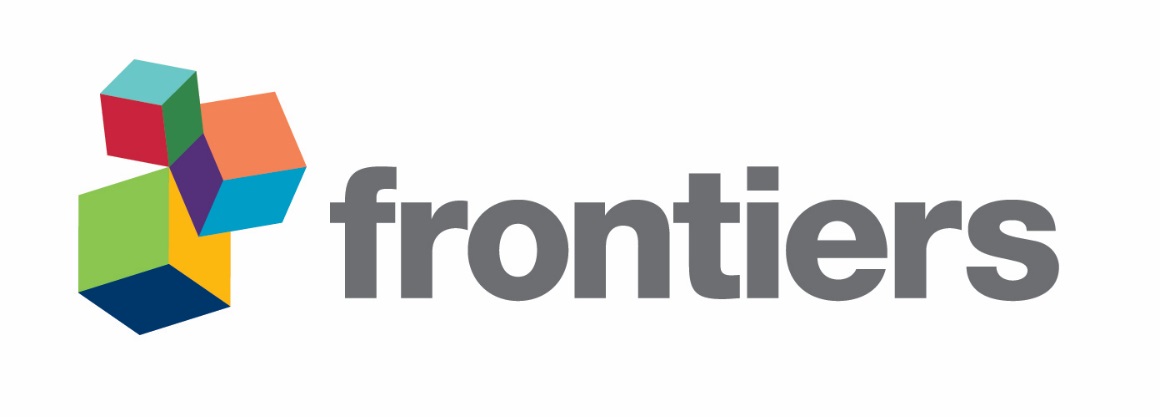
**

**Supplementary Table 4. Mean scores (SE) at the time points and differences (SE) between the pre-intervention and intervention periods (raw and**  **adjusted for covariates).**

|  | Mean (SE)^1^  t0 | Mean (SE)^1^  t1 | Mean (SE)^1^  t2 | Mean difference between periods | Adjusted^1^ mean difference between periods |
| --- | --- | --- | --- | --- | --- |
| **Burnout** | *n=65* | *n=357* | *n=276* |  |  |
| Burnout Measure total | 3.2 (0.07) | 3.2 (0.04) | 2.9 (0.04) | -0.3 (0.08)*** | -0.4 (0.09)*** |
|  |  |  |  |  |  |
| **Perceived stress** |  |  |  |  |  |
| PSQ Total (1-4) | 2.4 (0.04) | 2.4 (0.02) | 2.2 (0.02) | -0.2 (0.04)*** | -0.3 (0.05)*** |
| PSQ Demands (1-4) | 2.8 (0.06) | 2.8 (0.03) | 2.7 (0.03) | -0.2 (0.07)** | -0.3 (0.08) ** |
| PSQ Tension (1-4) | 2.5 (0.05) | 2.6 (0.03) | 2.3 (0.03) | -0.3 (0.06)*** | -0.4 (0.08)*** |
| PSQ Joy (1-4) | 2.6 (0.05) | 2.6 (0.03) | 2.8 (0.03) | 0.1 (0.06)* | 0.2 (0.07) * |
| PSQ Worry (1-4) | 2.0 (0.05) | 2.0 (0.03) | 1.8 (0.03) | -0.2 (0.06)*** | -0.3 (0.06) *** |
|  |  |  |  |  |  |
| **Mindfulness** |  |  |  |  |  |
| FMI Total (1-4) | 3.0 (0.05) | 2.8 (0.03) | 3.1 (0.03) | 0.4 (0.06)*** | 0.5 (0.07)*** |
| FMI Presence (1-4) | 3.0 (0.06) | 2.8 (0.03) | 3.2 (0.03) | 0.6 (0.07)*** | 0.6 (0.08)*** |
| FMI Acceptance (1-4) | 3.0 (0.05) | 2.9 (0.03) | 3.1 (0.03) | 0.3 (0.06)*** | 0.4 (0.07)*** |
|  |  |  |  |  |  |
| MAAS (1-4) | 2.4 (0.05) | 2.4 (0.02) | 2.6 (0.03) | 0.3 (0.06)*** | 0.4 (0.07)*** |
|  |  |  |  |  |  |
| **Well-being** |  |  |  |  |  |
| Well-being (1-7) | 4.4 (0.09) | 4.4 (0.05) | 4.8 (0.05) | 0.4 (0.1)*** | 0.6 (0.13)*** |
|  |  |  |  |  |  |
| **Organisational, team Climate, Personal performance** |  |  |  |  |  |
| Loti Cooperation (1-7) | 5.6 (0.06) | 5.4 (0.03) | 5.6 (0.03) | 0.3 (0.08)*** | 0.4 (0.09)*** |
| Loti Leadership (1-7) | 5.5 (0.08) | 5.5 (0.04) | 5.6 (0.05) | 0.1 (0.09) | 0.2 (0.10) |
| Loti Decision (1-7) | 5.2 (0.07) | 5.2 (0.04) | 5.4 (0.04) | 0.2 (0.08)** | 0.3 (0.10)** |
|  |  |  |  |  |  |
| Loti Respect (1-7) | 5.4 (0.07) | 5.4 (0.04) | 5.7 (0.04) | 0.3 (0.09)** | 0.3 (0.10)** |
| Loti Atmosphere (1-7) | 5.6 (0.07) | 5.6 (0.04) | 5.8 (0.04) | 0.2 (0.08)** | 0.3 (0.09)** |
|  |  |  |  |  |  |
| Loti Productivity (1-7) | 4.8 (0.09) | 4.7 (0.05) | 5.1 (0.05) | 0.5 (0.11)*** | 0.6 (0.12)*** |
| Loti Stress (1-7) | 3.7 (0.08) | 3.7 (0.04) | 3.3 (0.05) | -0.4 (0.09)*** | -0.5 (0.11)*** |

^*^ P <0.05 ^**^ P <0.01 ^***^ P <0.001

^1^ Adjusted for the covariates gender, company, leadership, children and living situation
